# [Retracted] miR-185-5p targets ROCK2 and inhibits cell migration and invasion of hepatocellular carcinoma

**DOI:** 10.3892/ol.2026.15822

**Published:** 2026-08-17

**Authors:** Yuexiang Niu, Gongen Tang

Oncol Lett 17: 5087–5093, 2019; DOI: 10.3892/ol.2019.10144

Following the publication of the above paper, it was drawn to the Editor's attention by a concerned reader that certain of the Transwell migration and invasion assay data shown in Figs. 2C, 4D and 5C and D had already appeared previously in a number of articles written by different authors at different departments/research institutes, one of which has subsequently been retracted.

Owing to the fact that the contentious data in the above article had already been either submitted or published prior to its submission to *Oncology Letters*, the Editor has decided that this paper should be retracted from the Journal. The authors were asked for an explanation to account for these concerns, but the Editorial Office did not receive a reply. The Editor apologizes to the readership for any inconvenience caused.

